# Comparison of Prior Bridging Intravenous Thrombolysis With Direct Endovascular Thrombectomy for Anterior Circulation Large Vessel Occlusion: Systematic Review and Meta-Analysis

**DOI:** 10.3389/fneur.2021.602370

**Published:** 2021-04-30

**Authors:** Zhao-Ji Chen, Xiao-Fang Li, Cheng-Yu Liang, Lei Cui, Li-Qing Yang, Yan-Min Xia, Wei Cao, Bu-Lang Gao

**Affiliations:** The Third ward of Neurology Department, Affiliated Hospital of Hebei University, Baoding, China

**Keywords:** bridging intravenous thrombolysis, endovascular thrombectomy, systematic review, anterior circulation, large vessel occlusion

## Abstract

**Background:** Whether bridging treatment combining intravenous thrombolysis (IVT) and endovascular thrombectomy (EVT) is superior to direct EVT alone for emergent large vessel occlusion (LVO) in the anterior circulation is unknown. A systematic review and a meta-analysis were performed to investigate and assess the effect and safety of bridging treatment *vs*. direct EVT in patients with LVO in the anterior circulation.

**Methods:** PubMed, EMBASE, and the Cochrane library were searched to assess the effect and safety of bridging treatment and direct EVT in LVO. Functional independence, mortality, asymptomatic and symptomatic intracranial hemorrhage (aICH and sICH, respectively), and successful recanalization were evaluated. The risk ratio and the 95% CI were analyzed.

**Results:** Among the eight studies included, there was no significant difference in the long-term functional independence (OR = 1.008, 95% CI = 0.845–1.204, *P* = 0.926), mortality (OR = 1.060, 95% CI = 0.840–1.336, *P* = 0.624), recanalization rate (OR = 1.015, 95% CI = 0.793–1.300, *P* = 0.905), and the incidence of sICH (OR = 1.320, 95% CI = 0.931–1.870, *P* = 0.119) between bridging therapy and direct EVT. After adjusting for confounding factors, bridging therapy showed a lower recanalization rate (effect size or ES = −0.377, 95% CI = −0.684 to −0.070, *P* = 0.016), but there was no significant difference in the long-term functional independence (ES = 0.057, 95% CI = −0.177 to 0.291, *P* = 0.634), mortality (ES = 0.693, 95% CI = −0.133 to 1.519, *P* = 0.100), and incidence of sICH (ES = −0.051, 95% CI = −0.687 to 0.585, *P* = 0.875) compared with direct EVT. Meanwhile, in the subgroup analysis of RCT, no significant difference was found in the long-term functional independence (OR = 0.927, 95% CI = 0.727–1.182, *P* = 0.539), recanalization rate (OR = 1.331, 95% CI = 0.948–1.867, *P* = 0.099), mortality (OR = 1.072, 95% CI = 0.776–1.481, *P* = 0.673), and sICH incidence (OR = 1.383, 95% CI = 0.806–2.374, *P* = 0.977) between patients receiving bridging therapy and those receiving direct DVT.

**Conclusion:** For stroke patients with acute anterior circulation occlusion and who are eligible for intravenous thrombolysis, there is no significant difference in the clinical effect between direct EVT and bridging therapy, which needs to be verified by more randomized controlled trials.

## Introduction

In the past 20 years, although intravenous thrombolysis (IVT) had been recognized as the most effective approach for acute ischemic stroke (AIS) (1), studies have shown that IVT presented inadequate response to emergent large vessel occlusion (ELVO) (2–4). Some landmark randomized clinical trials (RCTs) have provided some firm evidence that combining endovascular thrombectomy (EVT) with prior IVT (bridging treatment) is conspicuously superior to IVT alone (5–9). According to the above proof, the primary treatment strategy for ELVO in the anterior circulation is the bridging treatment recommended by the 2018 guidelines for the early treatment of patients with AIS by the American Heart Association/American Stroke Association (10). However, whether pretreatment with IVT is necessary is still debatable. Some studies hold the opinion that prior IVT was able to facilitate early recanalization and avoid further EVT (11–13). Additionally, pretreatment with IVT could soften the clot, thus shortening the EVT procedure, promoting the rate of mechanical recanalization, and reducing the number of passes of thrombectomy needed to achieve successful recanalization (11–13). On the contrary, some scholars thought that pretreatment with IVT would cause potential hemorrhagic complications, clot fragmentation, and distal embolization, possibly leading to a delay in the initiation of subsequent EVT (14–16). However, these concerns were only explored by retrospective analyses and observational studies, with low level of evidence. Moreover, some studies enrolled patients who were eligible for IVT into the direct EVT arm (17–20), whereas others enrolled patients who were not eligible for IVT into direct EVT treatment (21–24), which made these studies uncomparable. Recently, three RCTs have shown that bridging treatment was not superior to direct EVT for patients who were also eligible for IVT (25–27). Because of the controversies in this field regarding the application of bridging treatment (combining prior IVT and EVT) and direct EVT, a meta-analysis was needed to explore the advantages and disadvantages of both approaches of treatment in patients with IVT eligibility.

## Materials and Methods

### Search Strategy

This meta-analysis followed the PRISMA (Preferred Reporting Items for Systematic Reviews and Meta-Analyses) format guidelines (28). The protocol had been registered in the International System Evaluation Expected Register (PROSPERO; registration number CRD42020197147).

The electronic databases of PubMed, EMBASE, and Cochrane Library (deadline: May 22, 2020) were systematically searched using the title and the abstract retrieval method with no language restrictions: ((((((((Tissue Type Plasminogen Activator [Title/Abstract]) OR Plasminogen Activator, Tissue [Title/Abstract]) OR Alteplase [Title/Abstract]) OR Activase [Title/Abstract]) OR Tissue Plasminogen Activator [Title/Abstract]) OR thrombolysis [Title/Abstract])) AND ((((Thrombectomy[Title/Abstract]) OR mechanical thrombectomy [Title/Abstract]) OR endovascular reperfusion therapies [Title/Abstract]) OR endovascular treatment [Title/Abstract])) AND (((((acute ischemic stroke [Title/Abstract]) OR cerebral ischemia [Title/Abstract]) OR cerebral ischemia [Title/Abstract]) OR large vessel occlusion [Title/Abstract]) OR acute cerebral infarction [Title/Abstract]).

The selection criteria for articles eligible for this study were: (1) studies investigating patients who were older than 18 years; (2) studies with AIS patients of the anterior circulation; and (3) studies comparing outcomes between bridging (prior IVT + EVT) and direct EVT therapies. Articles which met the following exclusion criteria were excluded: (1) intra-arterial thrombolysis; (2) AIS of the posterior circulation; (3) lack of detailed information of patients with eligibility for IVT or EVT; and (4) patients ineligible for intravenous thrombolysis.

### Data Extraction and Assessment

Two researchers carefully read the literature, extracted the data, and reached an agreement on all items. Data obtained from the original studies were baseline data, primary outcome, and secondary outcomes. The primary outcome was functional independence (FI) used for assessing the efficacy of bridging treatment compared to direct EVT, which was defined as a modified Rankin Scale (mRS) score of 0–2 at 3 months. The secondary outcome included the long-term mortality defined as a mRS score of 6 at 90 days, successful reconstruction was defined as thrombolysis in cerebral infarction scores from 2b to 3, and safety outcomes were defined as symptomatic intracranial hemorrhage (sICH) and asymptomatic ICH (aICH) based on the criteria of the Heidelberg Bleeding Classification (8, 29). sICH refers to intracranial hemorrhage that compresses intact brain tissue, causing neurological symptoms and an increase in the NIHSS (National Institutes of Health Stroke Scale) score, with an increase of four or more points or an increase of two or more points of a NIHSS subcategory as a relevant change in neurological status, and potentially associated with a worsened long-term prognosis (29). Moreover, the mean time from hospital admission to groin puncture time (HTGT) was also analyzed. Subgroup analysis was used to explore the effect of different alteplase doses on the clinical outcomes (Supplementary Table 1). The data were adjusted for confounding factors before further analysis.

### Quality Evaluation of the Included Studies

The Newcastle-Ottawa Scale was used to evaluate the quality of the enrolled studies based on the selection of research population, comparability of establishment, and measurement of results (Supplementary Table 2) (30). After evaluation, eight articles with scores greater than or equal to 6 were considered high-quality articles, and one article with a score of <6 was considered a low-quality article and excluded (31). The publication bias of the studies included was categorized as high, low, or unclear risks and was assessed by two researchers (ZJC and CYL) independently according to the method provided in the study by Higgins et al. (32) including selection bias, performance bias, detection bias, attrition bias, reporting bias, and other potential biases. Any discrepancies were resolved by discussion or consultation with a third reviewer (XFL).

### Statistical Analysis

Statistical analysis was performed with the STATA software (version 14.0, Stata Corp. LP, College Station, TX, USA). A fixed-effects model pooled the data across trials and then compared with a random-effects model for the results. The odds ratio (OR) and standardized mean difference (SMD) with 95% confidence interval (CI) were used to evaluate the outcomes. Heterogeneity was assessed with the Cochran *Q* and *I*^2^ statistics. High heterogeneity was defined as *I*^2^ values ≥50%. Subgroup analysis was performed to evaluate the sources of heterogeneity. Publication bias was assessed using the Harbord's test with pseudo 95% confidence limits.

## Results

### Results of the Search and Characteristics of the Included Studies

**Figure 1:**
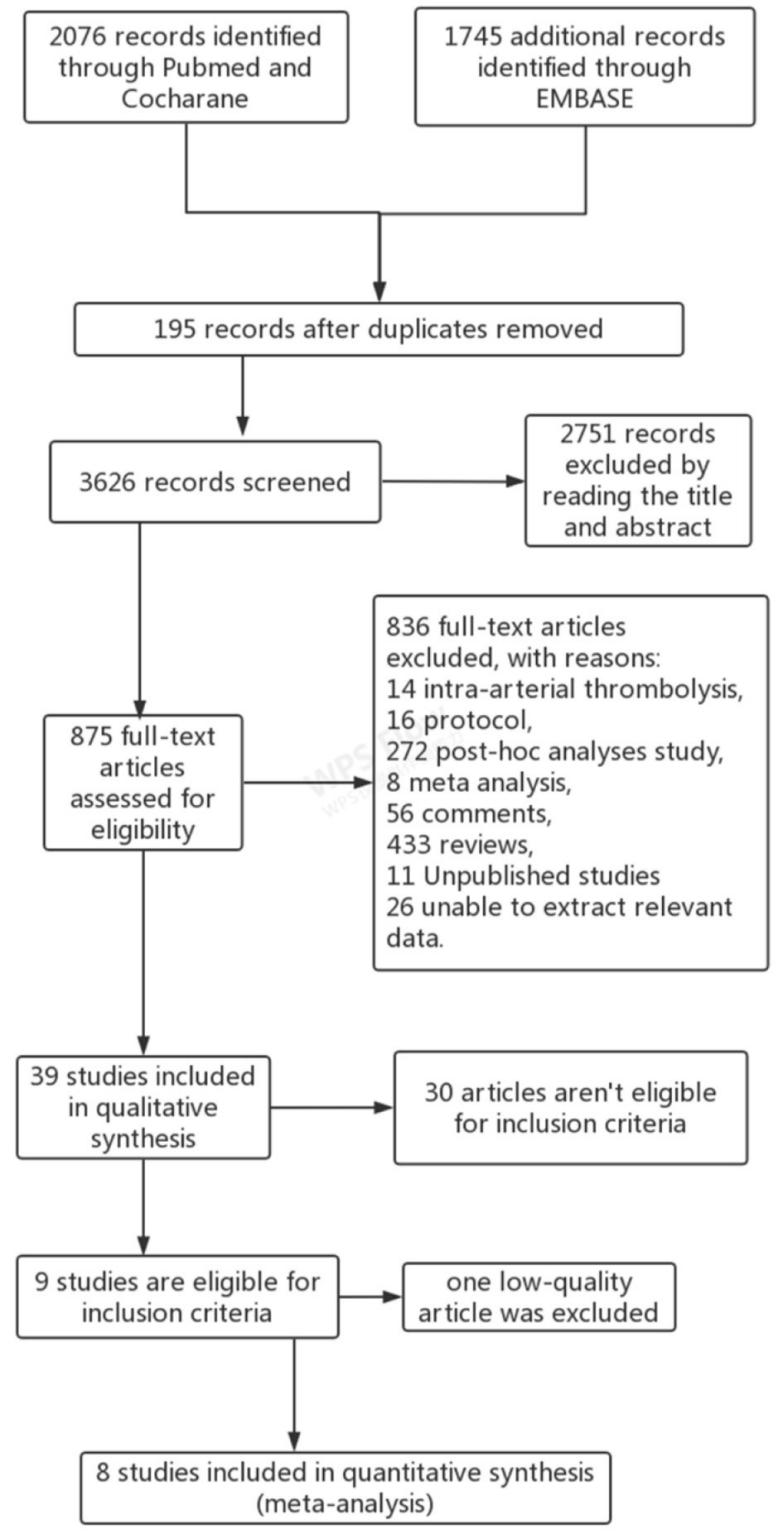
Flow diagram of study identification and selection.

A total of 3,821 articles were retrieved from three databases. After careful evaluation, 195 duplicate studies were excluded, 2,751 reports were eliminated because of improper titles and abstracts, and 836 articles were excluded due to the different reasons demonstrated in Figure 1. Among 39 studies in the qualitative synthesis, 21 full-text articles were further excluded mainly because these articles included patients with posterior circulation occlusion. Moreover, nine articles which included patients ineligible for IVT and one article with low quality were also excluded (31). Finally, eight articles met the inclusion criteria and were enrolled for analysis (Figure 1) (17–20, 25–27, 33). The flow diagram of the procedure for choosing the studies is shown in Figure 1, and the basic characteristics of the enrolled studies are shown in Table 1.

**Table 1:** Essential characteristics of the studies included in the meta-analysis.

**References**	**Country**	**Study type**	**Combined MT** **+** **IVT**					**Direct MT**					**Adjustment method**	**Follow-up** ** time**
			***N***	**Male (%)**	**Mean age**	**Baseline NIHSS**	**OTGT (min)**	***N***	**Male (%)**	**Mean age**	**Baseline NIHSS**	**OTGT (min)**		
Broeg-Morvay et al. (19)	Switzerland	Retrospective analysis	156	52.6%	73	17.7 ± 25.4	262.2 ± 85.2	40	62.5%	77	19.7 ± 26.1	228.6 ± 78.6	PS matching	90 days
Bellwald et al. (18)	Switzerland; Germany	Retrospective analysis	249	51%	73	16 (1–36)	4.26 ± 2.02	111	55.5%	75	15 (0–38)	3.23 ± 1.37	Multivariable regression	90 days
Wang et al. (20, 34)	China	Retrospective analysis	138	56.5%	67 (58.75–73)	17 (13–21.25)	Unknown	138	55.1%	67 (58.75–75)	16 (13–21)	Unknown	PS matching	90 days
Balodis et al. (17)	Latvia; UK	Prospective, observational study	84	45.2%	72	15 (12–18)	Unknown	62	45.2%	72	16.5 (14–20)	Unknown	Unknown	90 days
Gong et al. (33)	China	Retrospective analysis	42	64%	69	13 (6–21)	184.86 ± 56.8	31	48%	71	15 (6–22)	216.17 ± 88.3	PS matching	90 days
Yang et al. (25)	China	RCT	329	55%	69 (61–76)	17 (14–22)	Unknown	327	57.8%	69 (61–76)	17 (12–21)	Unknown	Multivariable regression	90 days
Suzuki et al. (26)	Japan	RCT	103	70%	76 (67–80)	17 (12–22)	Unknown	101	55%	74 (67–80)	19 (13–23)	Unknown	Unknown	90 days
Zi et al. (27)	China	RCT	118	55.9%	70 (60–78)	16 (13–20)	210 (179–255)	116	56.9%	70 (60–77)	16 (12–20)	200 (155–247)	Multivariable regression	90 days

*PS matching, propensity score matching*.

### Study Outcomes

#### Overall Results of the Pooled Data

**Figure 2:**
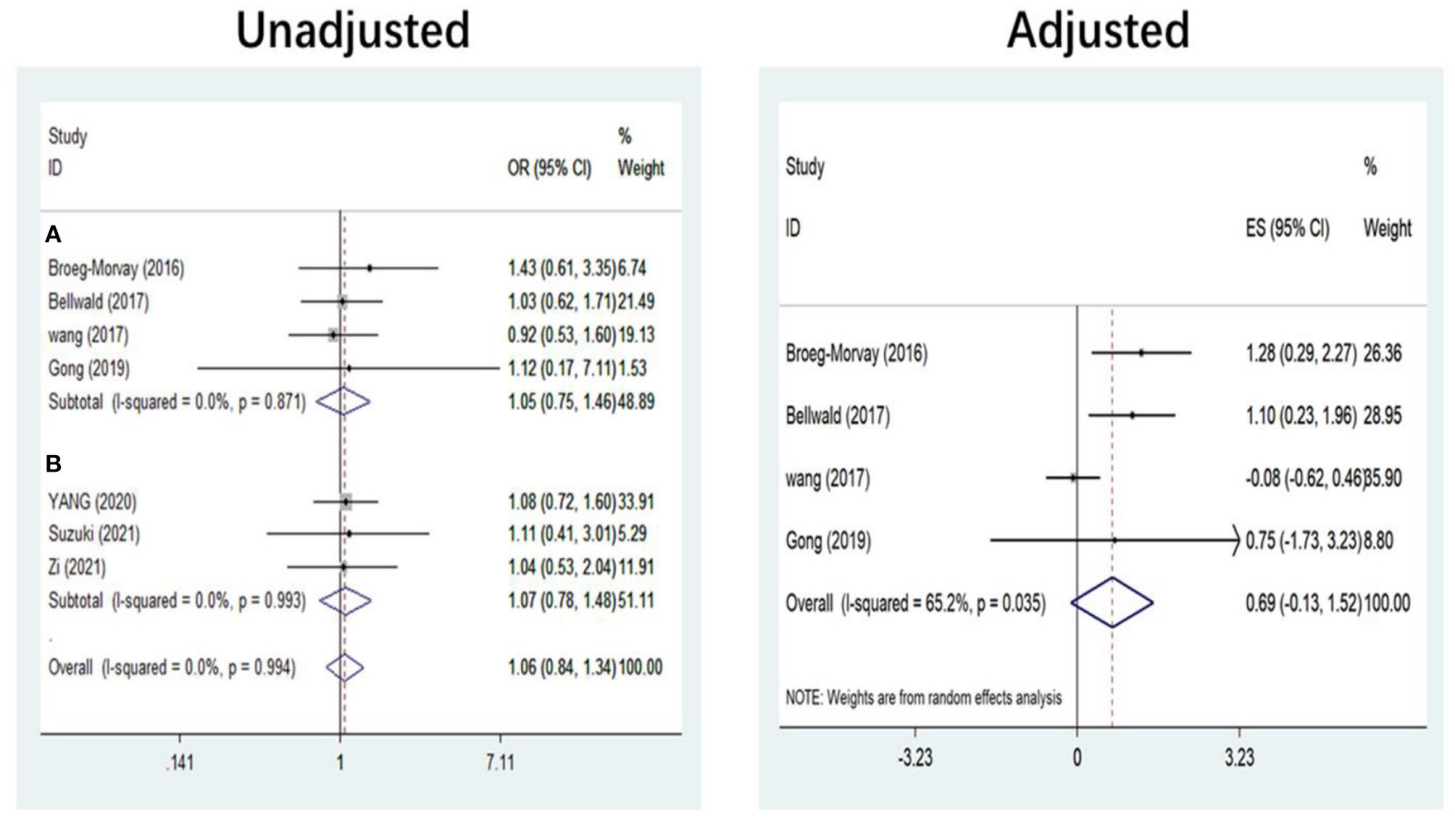
Mortality at 90 days. **(A)** Non-RCT. **(B)** RCT. *RCT*, randomized controlled trial; *ES*, effect size.

**Figure 3:**
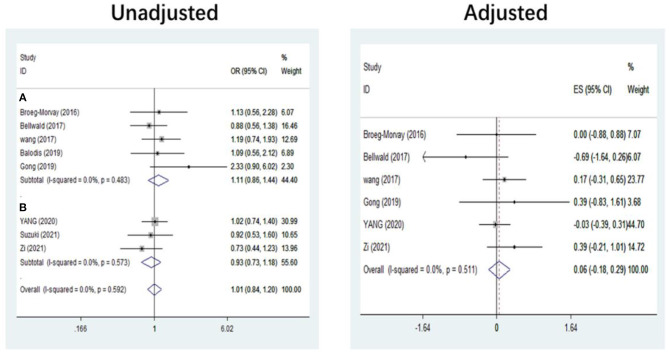
Functional independence at 90 days. **(A)** Non-RCT. **(B)** RCT. *RCT*, randomized controlled trial; *ES*, effect size.

**Figure 4:**
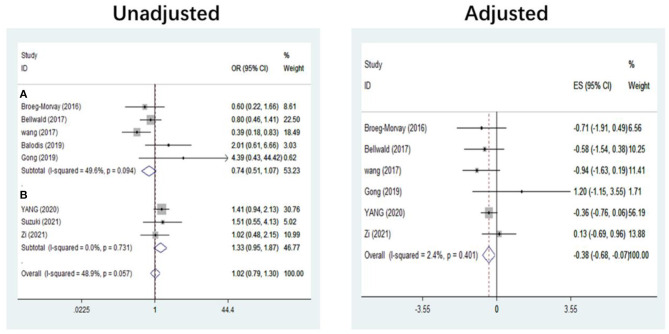
Recanalization. **(A)** Non-RCT. **(B)** RCT. *RCT*, randomized controlled trial; *ES*, effect size.

**Figure 5:**
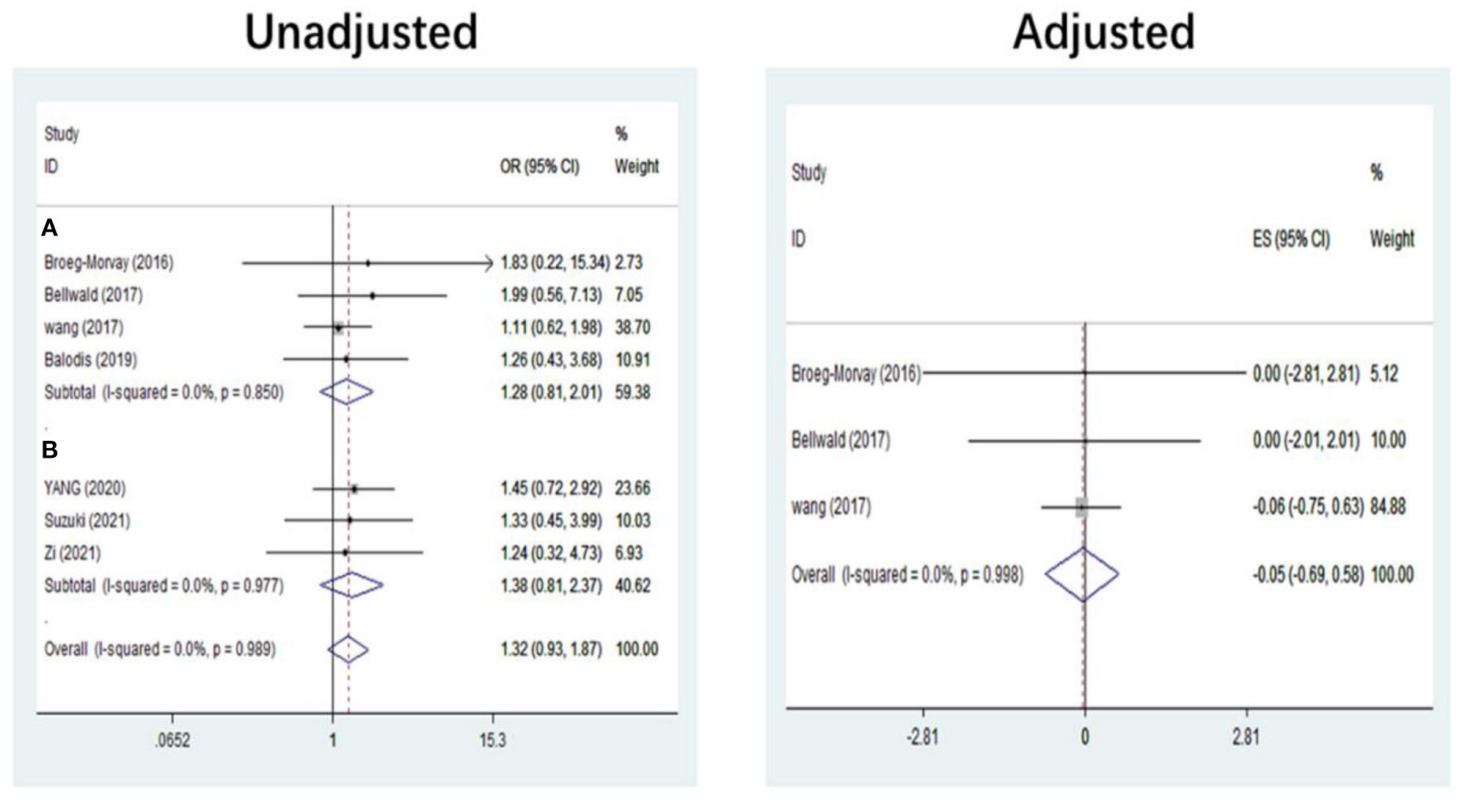
Symptomatic intracranial hemorrhage. **(A)** Patients ineligible for IVT. **(B)** Patients eligible for IVT. *IVT*, intravenous thrombolysis; *ES*, effect size.

The pooled results showed that bridging therapy was not superior to direct EVT. In terms of primary outcome, we found no significant difference in FI (OR = 1.008, 95% CI = 0.845–1.204, *P* = 0.926) (**Figure 3**) between bridging therapy and direct EVT. Meanwhile, there was no significant difference in the mortality rate at 90 days (OR = 1.060, 95% CI = 0.840–1.336, *P* = 0.624) (Figure 2), recanalization rate (OR = 1.015, 95% CI = 0.793–1.300, *P* = 0.905) (**Figure 4**), and risk of sICH (OR = 1.320, 95% CI = 0.931–1.870, *P* = 0.119) (**Figure 5**) between the two groups. However, bridging therapy significantly increased the risk of aICH compared to direct EVT (OR = 1.547, 95% CI = 1.242–1.927, *P* = 0.000) (Supplementary Figure 3). Interestingly, after adjustment for confounders, the vascular recanalization rate (effect size or ES = −0.377, 95% CI = −0.684 to −0.070, *P* = 0.016) (**Figure 4**) of bridging therapy was significantly lower than that of direct EVT. However, there was no significant change in FI (ES = 0.057, 95% CI = −0.177 to 0.291, *P* = 0.634) (Figure 3), mortality rate at 90 days (ES = 0.693, 95% CI = −0.133 to 1.519, *P* = 0.100) (Figure 2), and sICH (ES = −0.051, 95% CI = −0.687 to 0.585, *P* = 0.875) (**Figure 5**).

**Figure 6:**
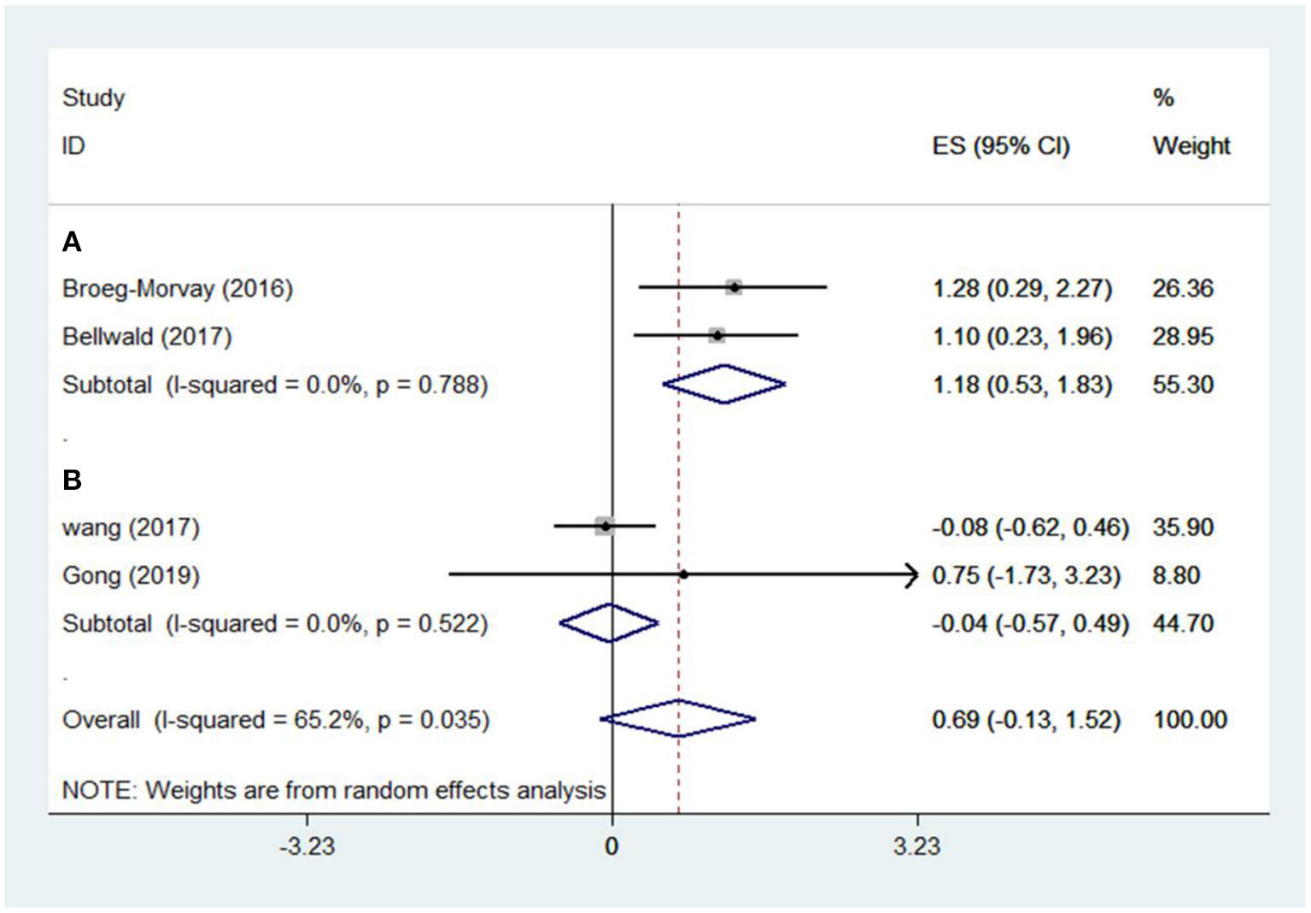
Adjusted mortality at 90 days. **(A)** Age > 70 years; **(B)** age ≤ 70 years. *ES*, effect size.

Significant heterogeneity was found in the outcome of adjusted mortality at 90 days (*I*^2^ = 65.2%, *P* = 0.035) (Figure 2), and age was a major source of heterogeneity by subgroup analysis. Compared with direct EVT, the long-term mortality in patients older than 70 years who received bridging therapy was significantly improved (OR = 1.178, 95% CI = 0.527–1.829, *P* = 0.000) (Figure 6).

### Subgroup Analysis of Study Type

Five non-RCT and three RCT studies were included in our study. In the non-RCT study, there was no significant difference in long-term mortality (OR = 1.047, 95% CI = 0.751–1.460, *P* = 0.787) (Figure 2), recanalization rate (OR = 0.750, 95% CI = 0.516–1.089, *P* = 0.131) (Figure 4), FI (OR = 1.111, 95% CI = 0.856–1.441, *P* = 0.428) (Figure 3), and sICH (OR = 1.276, 95% CI = 0.809–2.013, *P* = 0.850) (Figure 5) between bridging therapy and EVT, but bridging therapy had a significantly increased risk of aICH than EVT (OR = 2.069, 95% CI = 1.462–2.929, *P* = 0.000) (Supplementary Figure 3). In the RCT studies, bridging therapy did not have significant advantages in long-term mortality (OR = 1.072, 95% CI = 0.776–1.481, *P* = 0.673) (Figure 2), recanalization rate (OR = 1.331, 95% CI = 0.948–1.867, *P* = 0.099) (Figure 4), FI (OR = 0.927, 95% CI = 0.727–1.182, *P* = 0.539) (Figure 3), and risk of sICH (OR = 1.383, 95% CI = 0.806–2.374, *P* = 0.977) (Figure 5) in comparison with EVT. However, the pooled data from RCTs showed that the bridging treatment is more likely to improve the time from hospital admission to groin puncture than EVT, even though with no significant difference (OR = 0.101, 95% CI = −0.017 to 0.220, *P* = 0.095) (Supplementary Figure 2).

### Subgroup Analysis of Alteplase With Different Doses

In the bridging treatment group, two articles used either a two-third dose (0.6 mg/kg) or a full dose (0.9 mg/kg) of alteplase on the patients (18, 19), four articles used a full dose of alteplase (0.9 mg/kg) (17, 20, 25, 27), one article used a two-third dose (0.6 mg/kg) (26), and one article (33) did not describe the means of IVT (Supplementary Table 1). In the subgroup analysis, we mainly compared the patients' clinical outcomes who received either a two-third dose or a full dose of alteplase with those who only received a full dose of alteplase (Supplementary Table 3). Although the results showed that there are no significant difference between the two subgroups, the tendency of better clinical outcomes was found for the study using the full dose of alteplase than the study adopting the different dose of alteplase, with a lower mortality at 90 days (OR = 1.025, 95% CI = 0.767–1.370), higher FI at 90 days (OR = 0.998, 95% CI = 0.800–1.246), a higher recanalization rate (OR = 1.075, 95% CI = 0.791–1.461), and a lower risk of sICH (OR = 1.242, 95% CI = 0.838–1.840).

### Sensitivity, Publication Bias, and Quality of the Studies

Based on the analysis using the Newcastle-Ottawa Scale (30), eight studies were of high quality while one was of low quality. The primary outcomes were tested with the Harbord statistical test, with no significant (*P* > 0.1) publication bias detected (Supplementary Figure 5). To investigate the stability of the primary outcomes, a sensitivity analysis was further performed, and no studies were found to significantly affect the outcomes (Supplementary Figure 4).

## Discussion

In this meta-analysis, five retrospective studies and three RCTs were included. The pooled results showed that bridging therapy was not superior to direct EVT in patients who underwent direct EVT with ELVO of the anterior circulation; however, bridging therapy increased the risk of aICH compared with direct EVT. After adjusting for confounding factors, bridging therapy showed a significantly lower recanalization rate than direct EVT. Moreover, the pooled results from the RCTs demonstrated that patients eligible for IVT who received direct EVT showed no significant difference in long-term mortality, FI, recanalization rate, and incidence of sICH compared with those who underwent the bridging treatment.

Some meta-analyses had also compared direct EVT and bridging treatment (35–38). In comparison with patients who experienced mechanical thrombectomy (MT) only in the study by Mistry et al. (37) patients receiving MT + IVT had better functional outcomes (mRS score = 0–2, summary OR = 1.27, 95% CI = 1.05–1.55, *P* = 0.02), lower mortality (OR = 0.71, 95% CI = 0.55–0.91, *P* = 0.006), and a higher rate of successful recanalization (OR = 1.46, 95% CI = 1.09–1.96, *P* = 0.01). A greater number of MT + IVT patients required two or fewer passes with a neurothrombectomy device to achieve successful recanalization (OR = 2.06, 95% CI = 1.37–3.10, *P* = 0.0005). In the meta-analysis by Pan et al. (38) significantly more patients who received MT + IVT obtained higher functional independence (OR = 1.21, 95% CI = 1.13–1.30) and successful recanalization rate (OR = 1.09, 95% CI = 1.02–1.15) than patients with MT alone. However, the pooled results showed a significantly lower mortality (OR = 0.74, 95% CI = 0.66–0.83) for patients who received MT + IVT. In the study by Liu and Li (36), compared with patients who received bridging treatment, patients treated with direct EVT had a similar likelihood of achieving good functional outcome at 3 months (OR = 0.93, 95% CI = 0.85–1.01, *P* = 0.094), similar mortality at 3 months (OR = 1.10, 95% CI = 0.91–1.33, *P* = 0.33), and sICH (OR = 1.06, 95% CI = 0.74–1.51, *P* = 0.75), with a lower risk of intracranial hemorrhage (OR = 0.76, 95% CI = 0.60–0.95, *P* = 0.02). In the meta-analysis by Kaesmacher et al. (35) the rate of successful reperfusion was not significantly different in patients receiving direct EVT and bridging treatment (OR = 0.93, 95% CI = 0.68–1.28). In studies including IVT-ineligible patients in direct EVT, patients undergoing direct EVT tended to have lower rates of functional independence and higher odds of a fatal outcome as compared to patients with bridging treatment (OR = 0.78, 95% CI = 0.61–1.01 and OR = 1.45, 95% CI = 1.22–1.73, respectively). However, no such treatment effect was found when the analyses were confined to cohorts with a lower risk of selection bias (including IVT-eligible direct EVT patients). The results of the meta-analyses by Mistry et al. (37) and Pan et al. (38) were not consistent with the conclusion of our study because they enrolled patients ineligible for IVT in the direct EVT group, but without further subgroup analysis, thus resulting in a conclusion favorable for IVT. The results of the meta-analyses by Liu and Li (36) and Kaesmacher et al. (35) were consistent with our conclusion; however, the study by Liu and Li. (36) did not investigate other variables except FI, besides enrollment of patients with large vessel occlusion in the posterior circulation. Kaesmacher et al. (35) studied patients eligible for IVT and were of the opinion that direct EVT did not have significantly different effects from bridging treatment; however, only three studies enrolled patients eligible for IVT. Moreover, their study (35) also enrolled patients with large vessel occlusion in the posterior circulation. Thus, these meta-analyses were uncomparable to ours, which enrolled only patients with ELVO in the anterior circulation.

There are currently three RCTs suggesting that there was no significant difference between direct EVT and bridging therapy in stroke patients with acute anterior circulation occlusion of the great vessels and who are eligible for IVT (25–27). Two RCT trials from China have confirmed the non-inferiority of EVT alone *vs*. bridging therapy in long-term functional independence (mRS score of 0–2) (25, 27). However, this conclusion was not confirmed in a study from Japan (26). It is worth noting that the dosage of alteplase used in the bridging group in the study by Suzuki et al. is 0.6 mg/kg (26), which is inconsistent with the standard dosage recommended by the latest guidelines (10). In addition, Suzuki et al. also mentioned in the article that the non-inferiority margin was calculated based on the data of full-dose alteplase (0.9 mg/kg), which may lead to errors in the recent research results (26). Actually, in our included studies, different therapeutic doses of alteplase were used in the bridging group of some studies, including full dose (0.9 mg/kg) (17, 25, 27, 34) and two-third dose (0.6 mg/kg) (26) of alteplase; the two doses were present at the same time (18, 19) (Supplementary Table 1). Based on these limited data, we can only discuss the clinical outcomes of all patients receiving full-dose alteplase and those receiving different doses of alteplase. Our study revealed the tendency of better clinical outcomes for the studies using the full dose of alteplase than those adopting different doses of alteplase (Supplementary Table 3).

In terms of adverse events, Zi et al. reported that patients in the bridging group had a higher risk of aICH (15.7 *vs*. 25.6%) (27), which is consistent with our conclusion. Suzuki et al. reported that patients in the bridging group had a significantly increased rate of any ICH at 36 h from onset (33.7 *vs*. 50.5%) (26). However, Yang et al. did not find any significant difference in the bleeding events between the two groups (25), partly because 30 patients (9.1%) in the bridging group did not receive a full dose (*n* = 20) or any dose (*n* = 10) of alteplase, whereas 37 patients (11.2%) did not receive thrombectomy. In our study, we mainly evaluated the risk of patients with sICH and aICH, and the results showed that the risk of patients with aICH was significantly higher with bridging therapy. Current studies have suggested that increased bleeding events after endovascular treatment are associated with poor clinical outcomes (39, 40). However, due to the different doses of alteplase used in some studies, our conclusion needs further confirmation.

Unfortunately, due to limited data available, more clinical outcomes could not be analyzed between the two treatment approaches. Although our study found no significant difference in the time from hospital admission to groin puncture in RCTs between the two groups of therapy, bridging therapy tended to prolong the puncture time. In addition, Suzuki's research mentioned 22 patients (21.4%) in the bridging treatment group who had groin puncture before receiving intravenous thrombolytics (26). It was further pointed out that recombinant tissue plasminogen activator (rt-PA) administration might not be a disadvantage to the starting of mechanical thrombosis. In fact, in the real world, bridging therapy may be more complicated, and patients may have already received drug thrombolytic therapy before presentation to the hospital for mechanical thrombosis, making bridging therapy even longer. The STRATIS Study found that patients with interrupted bridging therapy experienced a 124-min difference between interhospital transfer therapy and direct in-hospital treatment and had better clinical outcomes (mRS = 0–1) (48.7 *vs*. 39.9%, OR = 1.43, 95% CI = 1.03–1.99, *P* = 0.04) (41). This conclusion is consistent with the results from the HERMES meta-analysis and a real-world data from Melbourne (42, 43). Unfortunately, none of the studies included in our study explored this question. In addition, the pre-hospital management systems in different countries are not consistent, resulting in limited data and outcomes in this area available for analysis (25).

Interestingly, the recanalization rate in the bridging treatment was lower after than before adjustment for confounders, which was probably caused by the exclusion of patients who had achieved early successful recanalization following prior IVT in some studies (18, 19, 33). Early recanalization caused by pretreatment with IVT was non-negligible in real life and was probably the advantage of prior IVT. However, our meta-analysis did not include any case with early recanalization. There was only one study reporting pretreatment with IVT in patients with ELVO eligible for EVT that resulted in successful recanalization in 1 of 10 cases (44). It is worth noting that the efficacy of a new thrombolytic drug, tenecteplase, a genetically modified variant of alteplase for promoting early recanalization of great vessel occlusion, has been the focus of attention since its introduction (44). Compared with alteplase, tenecteplase has the fibrin specificity increased by 14 times, the fibrinogen preservation rate increased by 10 times, and the resistance to plasminogen activator inhibitor-1 activity increased by 80 times, with faster thrombolysis, reduced plasma clearance rate, longer half-life, and convenient administration (45). Tenecteplase has been compared with alteplase in a large number of clinical trials; however, the advantage of tenecteplase in early recanalization of large vessel occlusion was only confirmed in some RCTs with small samples (46, 47). In addition, other studies reported the inconsistent efficacy of tenecteplase (48–50). The latest RCT showed that compared with alteplase, tenecteplase significantly increased the early recanalization rate (22 *vs*. 10%) and reduced the long-term mortality (10 *vs*. 18%) in stroke patients with large vessel occlusion, but had no significant difference in the bleeding risk (51). However, it should also be noted that the final recanalization rate was not significantly different (52 *vs*. 57%) between tenecteplase and alteplase. In addition, due to the small sample size in the study, the long-term mortality rate still needs to be confirmed in large samples of RCTs.

Stent retrievers have been recommended for mechanical thrombectomy in the 2015 and 2018 AHA–ASA guidelines (10, 52). Among our studies, five articles mentioned the combination of aspiration and stent recovery technology in the EVT arm (17, 25–27, 33), one study only used stent recovery technology (19), and the remaining two did not mention this technology at all (18, 20). At present, the 2018 AHA–ASA guideline still recommended the stent recycling technology, pointing out that there was no significant difference between aspiration and stent recycling technology (10). However, insufficient information was obtained from the enrolled studies in our meta-analysis about endovascular treatment using the stent technology; it was thus impossible to evaluate the outcomes of stent aspiration and mechanical thrombectomy.

Some limitations exist in our study. There were only three RCTs; all other articles were of a retrospective nature. More RCT studies are needed. Furthermore, some studies had high heterogeneity whose source was not found due to limited data. The currently enrolled limited studies restricted the coverage of all clinical outcomes. Future studies will have to resolve all these issues for better outcomes.

In conclusion, in patients with acute anterior occlusion and IVT compliance, direct DVT and bridging therapy have no significant prognostic difference, but bridging therapy may significantly increase the risk of asymptomatic intracranial hemorrhage.

## Data Availability Statement

The raw data supporting the conclusions of this article will be made available by the authors, without undue reservation.

## Author Contributions

Z-JC, X-FL, and B-LG: study design and data analysis. Z-JC, C-YL, LC, L-QY, Y-MX, and WC: data collection. Y-MX and WC: study supervision. All authors: approval of the study.

## Conflict of Interest

The authors declare that the research was conducted in the absence of any commercial or financial relationships that could be construed as a potential conflict of interest.

## Abbreviations

IVT: intravenous thrombolysis
AIS: acute ischemic stroke
ELVO: emergent large vessel occlusion
RCT: randomized clinical trial
EVT: endovascular thrombectomy
mRS: modified Rankin Scale
sICH: symptomatic intracranial hemorrhage
aICH: asymptomatic intracerebral hemorrhage
OTGT: mean time from onset to groin puncture time
PL: procedure length
NI: neurologic improvement
OR: odds ratio
SMD: standardized mean difference
CI: confidence interval
NIHSS: National Institutes of Health Stroke Scale
FI: functional independence
ES: effect size.
