# Supplementary material for: Comparison of Prior Bridging Intravenous Thrombolysis With Direct Endovascular Thrombectomy for Anterior Circulation Large Vessel Occlusion: Systematic Review and Meta-Analysis
Source: Front Neurol. 2021 Apr 30;12:602370. doi: 10.3389/fneur.2021.602370 (PMC8120007; doi:10.3389/fneur.2021.602370)
Supplement: Supplementary file 1 [file Data_Sheet_1.doc]

**Supplemental data**

**Supplemental tables**

**Supplemental Table 1.** Results of 8 studies included in the study

| Study | Year | aICH | Dose of alteplase | HTGT |
| --- | --- | --- | --- | --- |
| Broeg-Morvay^19^ | 2016 | √ | 0.9 mL/kg or  0.6 mL/kg | \ |
| Wang^20^ | 2017 | √ | 0.9 mL/kg | \ |
| Bellwald^18^ | 2017 | √ | 0.9 mL/kg or  0.6 mL/kg | \ |
| Balodis^17^ | 2019 | √ | 0.9 mL/kg | \ |
| Gong^33^ | 2019 | \ | unknown | \ |
| Yang^25^ | 2020 | √ | 0.9mg/kg | √ |
| Zi^27^ | 2021 | \ | 0.9mg/kg | √ |
| Suzuki^26^ | 2021 | \ | 0.6-mg/kg | √ |

Note: √, positive in the study; \, negative in the study; aICH, asymptomatic intracerebral hemorrhage; HTGT, mean time from hospital admission to groin puncture; superscript number indicate the reference number.

**Supplemental Table 2.** Quality of 9 studies evaluated with the Newcastle-Ottawa Scale

| **Study name**  **Year** | **Selection** | **Comparability** | **Outcome** | **Overall score** |
| --- | --- | --- | --- | --- |
| Broeg-Morvay et al, 2016^19^ | 3 | 2 | 3 | 8 |
| Bellwald et al, 2017^18^ | 3 | 2 | 2 | 7 |
| Wang et al, 2017^20^ | 4 | 2 | 3 | 9 |
| Balodis et al, 2018^17^ | 3 | 0 | 3 | 6 |
| Gong et al, 2019^33^ | 2 | 2 | 3 | 7 |
| Casetta et al, 2019^31^ | 1 | 2 | 2 | 5 |
| Yang et al, 2020^25^ | 4 | 2 | 3 | 9 |
| Suzuki et al, 2021^26^ | 4 | 1 | 4 | 9 |
| Zi et al, 2021^27^ | 3 | 2 | 4 | 9 |

Note: Superscript number indicate the reference number. The study by Casetta et al, 2019^31^ was excluded due to low to a low quality score of 5 (less than 6).

**Supplemental Table 3.** Subgroup analysis on the effect of alteplase

| Clinical outcome | A (OR(95%CI)) | B(OR(95%CI)) | Overall (OR(95%CI)) | P |
| --- | --- | --- | --- | --- |
| Mortality at 90 days | 1.126(0.731,1.734) | 1.025(0.767,1.370) | 1.056(0.830,1.342) | 0.66 |
| FI 90 days | 0.946(0.647,1.382) | 0.998(0.800,1.246) | 0.985(0.813,1.192) | 0.88 |
| Recanalization | 0.768(0.470,1.254) | 1.075(0.791,1.461) | 0.975(0.753,1.263) | 0.85 |
| sICH | 1.946(0.651,5.815) | 1.242(0.838,1.840) | 1.318(0.913,1.904) | 0.14 |

Note: A, patients received either 2/3 dose of altiplase or full dose of altiplase; B, studies using full dose of altiplase; FI, functional independence; sICH, symptomatic intracranial hemorrhage; OR, odds ratio; Overall, combination of groups A and B; CI, confidence interval.

**Supplemental figures and legends**


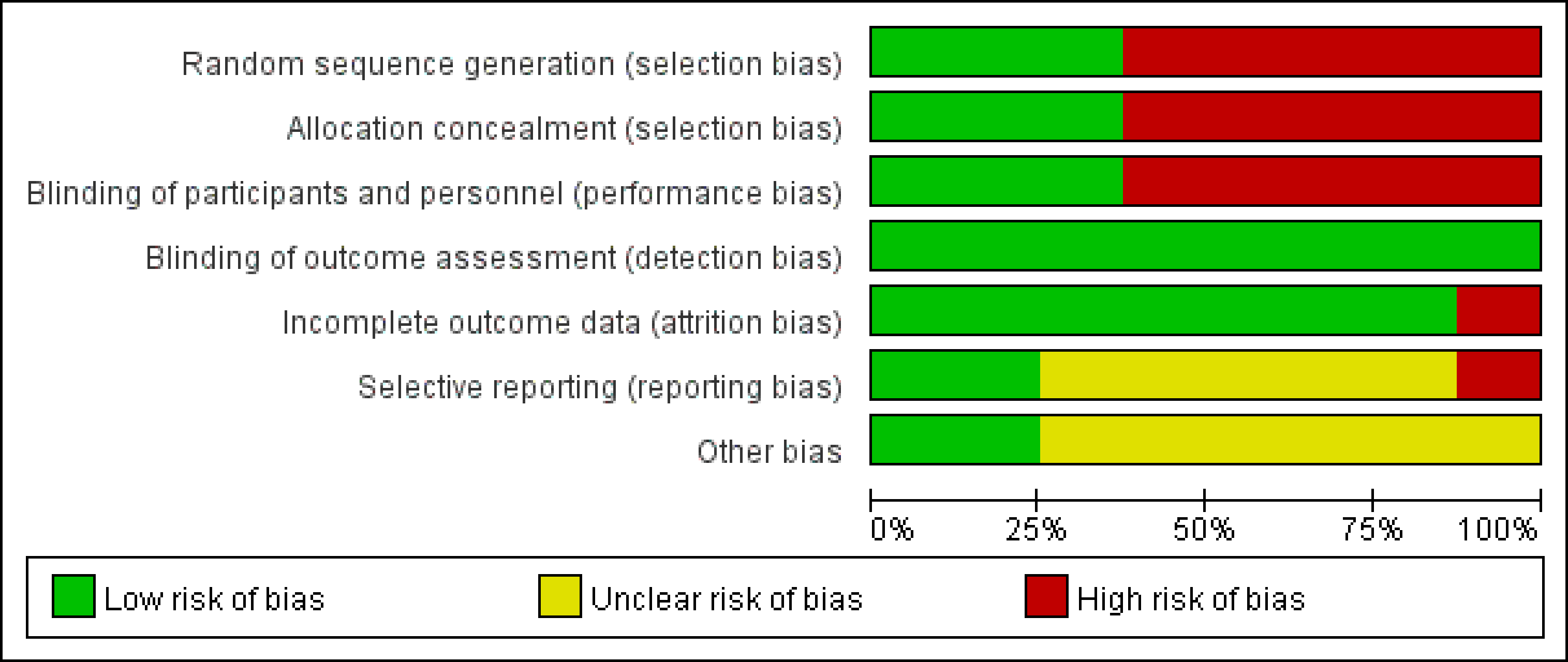


**Supplemental Fig 1.** Assessment of study bias.


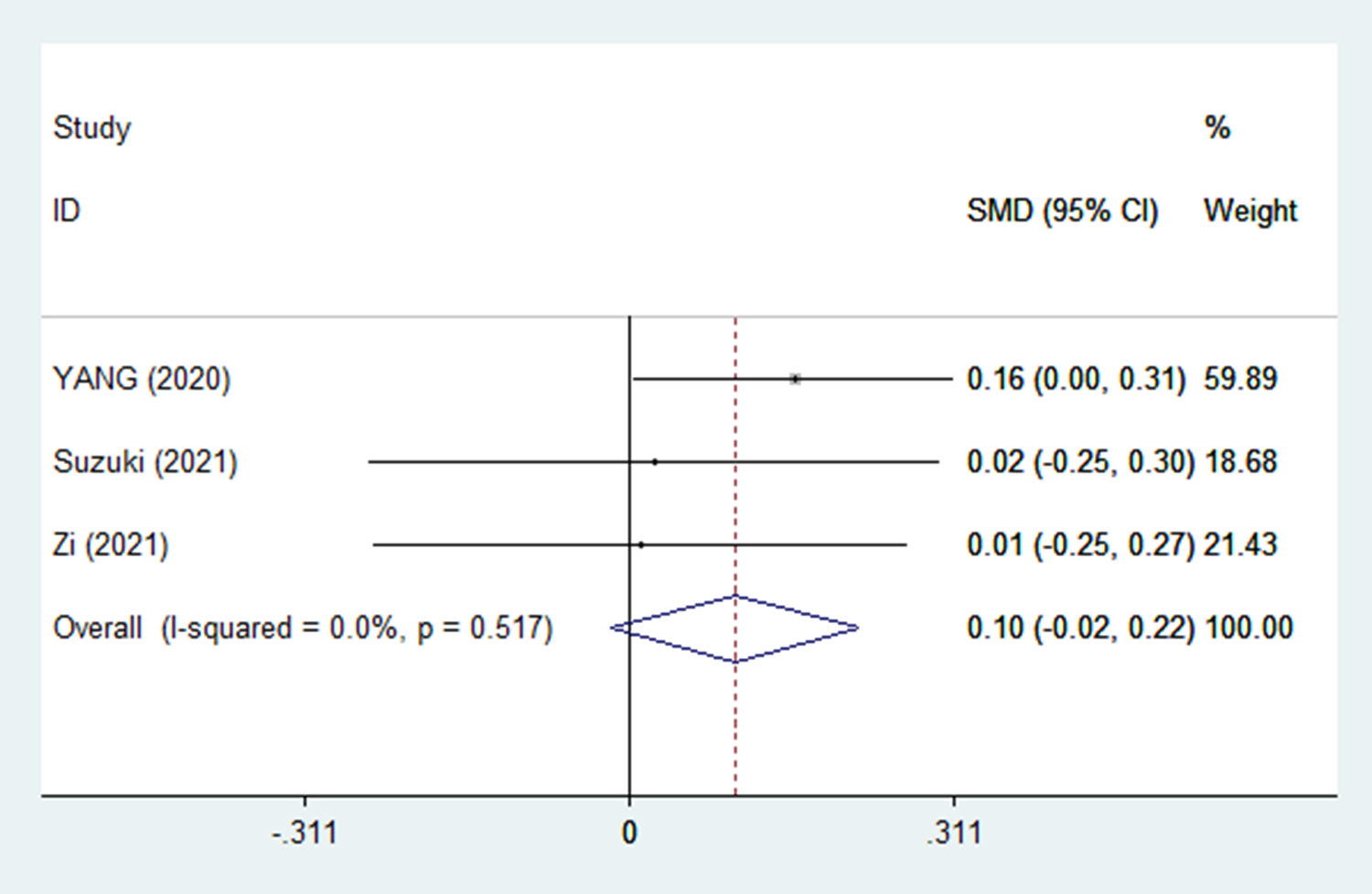


**Supplemental Fig 2.** From hospital admission to groin puncture.


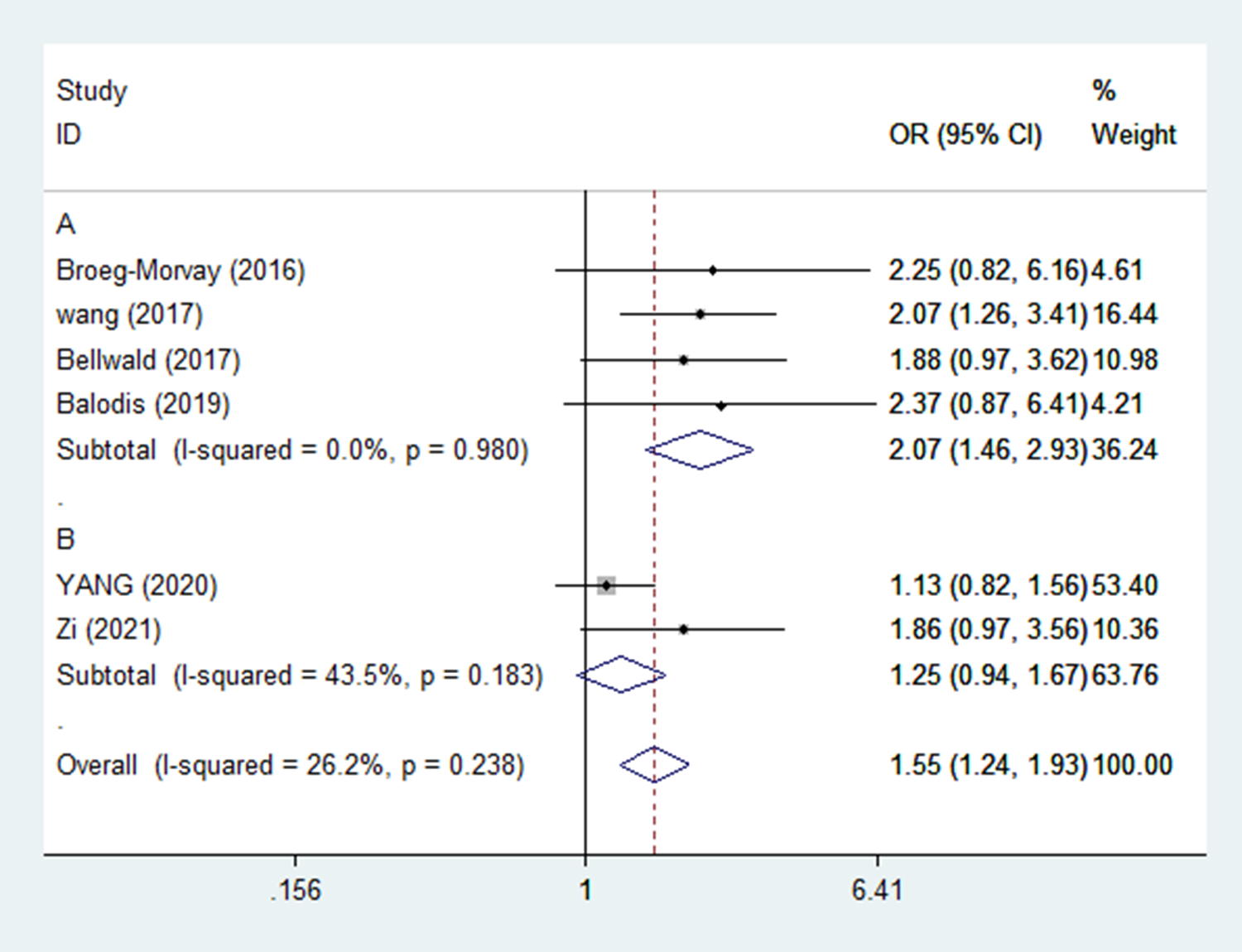


**Supplemental Fig 3.** asymptomatic intracerebral hemorrhage. A: non-RCT; B: RCT. RCT, [randomized](javascript:;) [controlled](javascript:;) [trial](javascript:;).


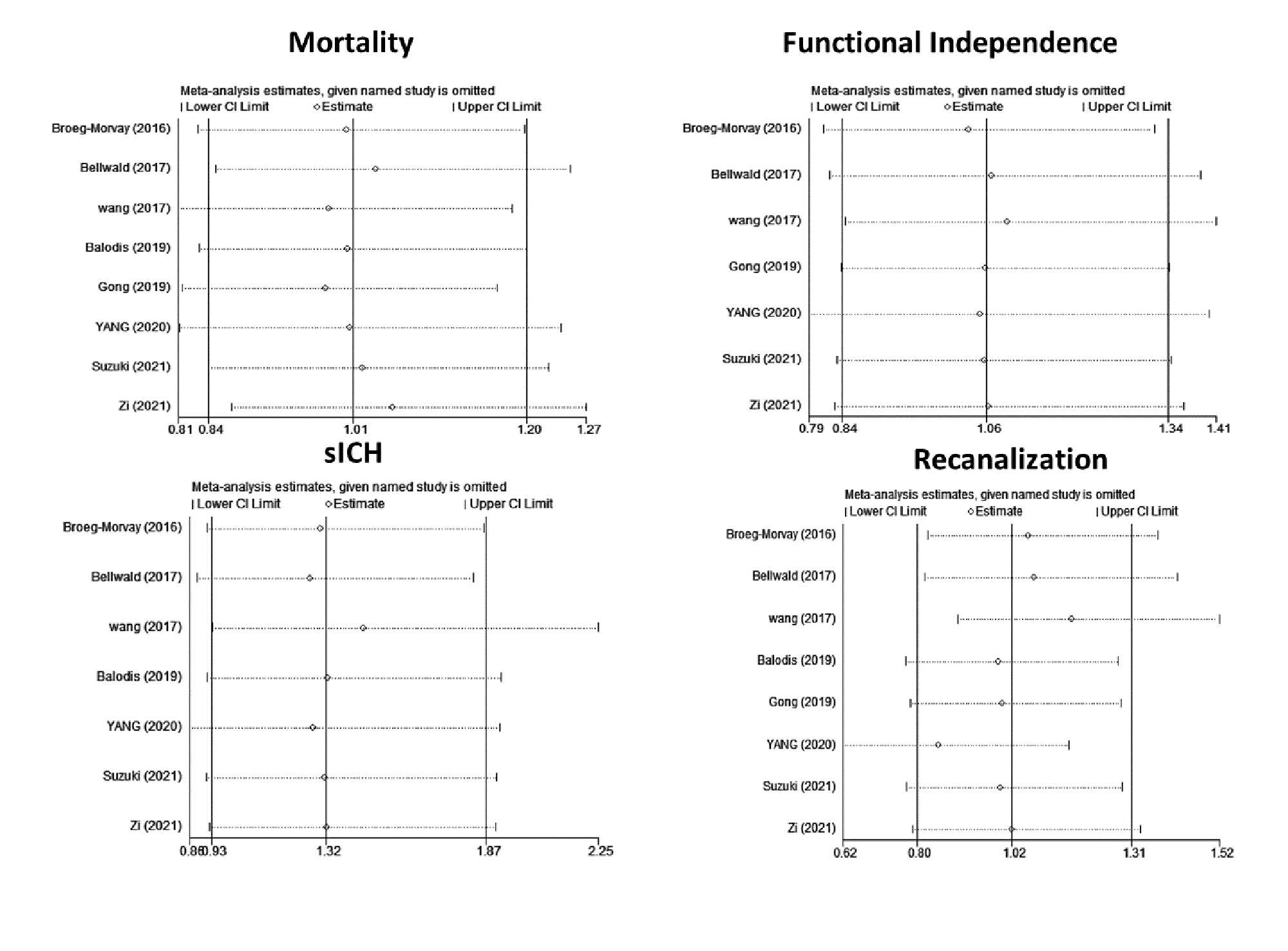


**Supplementary Fig 4.** Sensitivity Analysis


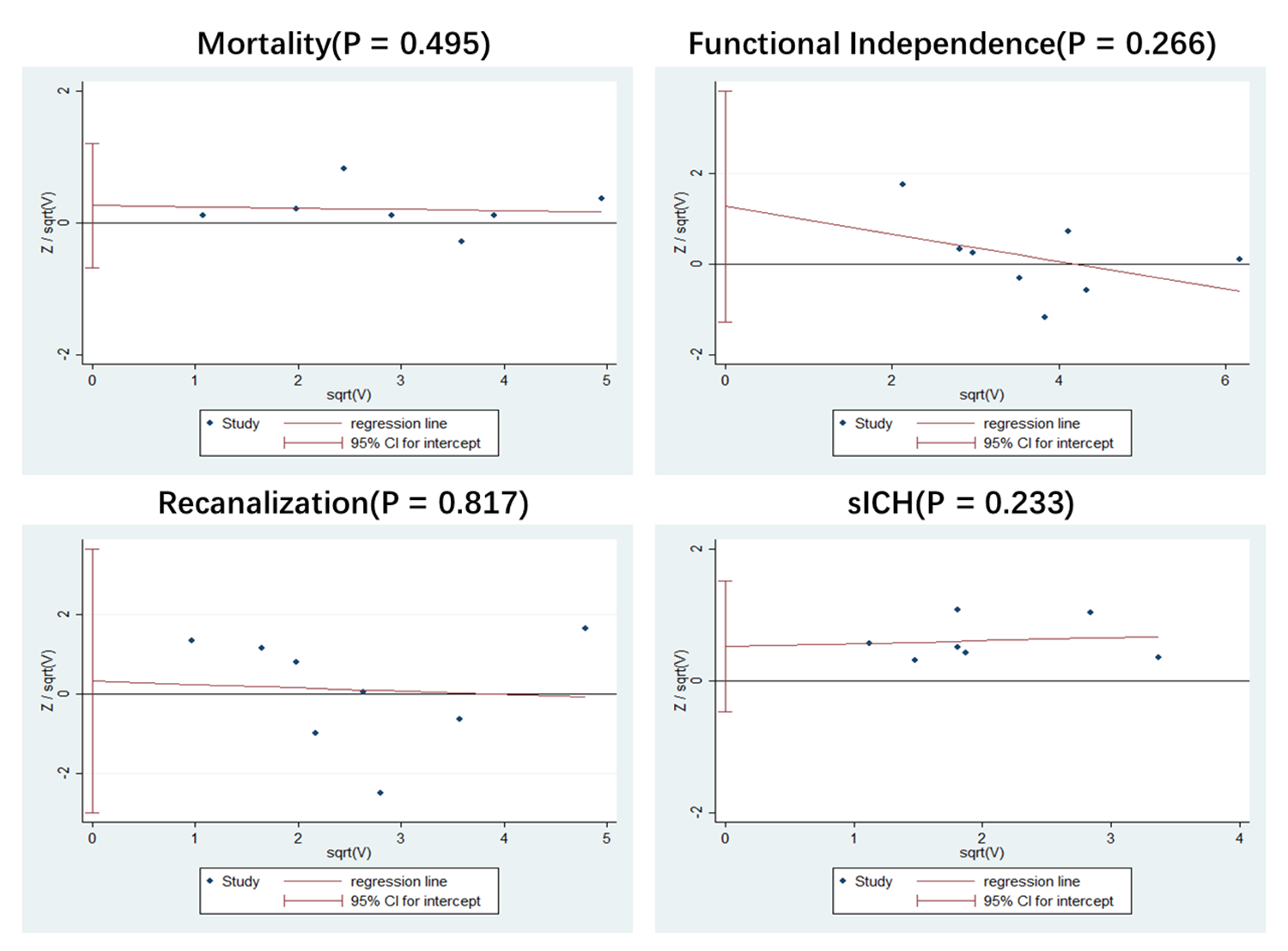


**Supplemental Fig 5.** Detection of the bias publication
